# Prediction of germline BRCA mutation using clinicopathologic, MRI semantic, and radiomics features in high-risk breast cancer patients: a multicenter study

**DOI:** 10.3389/fradi.2026.1858292

**Published:** 2026-06-19

**Authors:** Yoon Sang Cho, Eunje Oh, Yoo Jin Han, Kyu Ran Cho, Kyong Hwa Park, Sung Eun Song

**Affiliations:** 1 Advanced Medical Imaging Institute, Korea University Anam Hospital, Korea University College of Medicine, Seoul, Republic of Korea; 2 Korea University Anam Hospital, Korea University College of Medicine, Seoul, Republic of Korea; 3 Department of Radiology, Korea University Anam Hospital, Korea University College of Medicine, Seoul, Republic of Korea

**Keywords:** breast MRI, clinical features, external validation, germline BRCA mutation, high-risk breast cancer, machine learning, radiomics

## Abstract

**Background and purpose:**

BRCA mutations are strongly associated with hereditary breast cancer and have important implications for personalized treatment; however, genetic testing may be costly. This highlights the need for noninvasive, practical approaches to prioritize patients most likely to benefit from confirmatory testing. This study evaluated the predictive value of clinicopathologic features, radiologist-assessed magnetic resonance imaging (MRI) semantic features, MRI-derived radiomics features, and their multimodal integration for identifying germline BRCA mutation status.

**Patients and methods:**

This retrospective multicenter study included high-risk breast cancer patients from two institutions (Center A and Center B) who underwent preoperative breast MRI and germline BRCA testing. Three types of predictors were used: clinicopathologic features, radiologist-assessed MRI semantic features, and MRI-derived radiomic features. Radiomics features were extracted from tumor masks on 2-minute contrast-enhanced subtraction images and T2-weighted images of preoperative MRI using a standardized, open-source PyRadiomics pipeline. Six machine learning models, including logistic regression (LR), random forest (RF), support vector machine (SVM), gradient-boosted models (LightGBM and XGBoost), and multilayer perceptron (MLP), were performed using unimodal models and their multimodal combinations. Model performance was evaluated using the area under the receiver operating characteristic curve (AUC) in both internal validation (10 repeated random train-test splits in Center A) and external cross-center validation (training on Center A and testing on Center B).

**Results:**

A total of 492 patients were included (Center A, *n* = 270; Center B, *n* = 222). In internal validation, clinicopathologic-based LR model (AUC = 0.73) and radiomics (sub-T1WI)-based gradient-boosting models (LightGBM and XGBoost) achieved the highest performance (AUC = 0.71 and 0.72, respectively). In external validation, the clinicopathologic-based RF model (AUC = 0.73) and the combined clinicopathologic and radiologist-assessed MRI-based RF model (AUC = 0.77) achieved the highest performance. The multimodal features integrating clinicopathologic, radiologist-assessed MRI, and sub-T1WI radiomics features-based LR model (AUC = 0.72) achieved comparable performance.

**Conclusions:**

A non-invasive machine learning model integrating clinicopathologic, radiologist-assessed MRI features, and radiomic signatures provides complementary predictive information for germline BRCA mutation status in high-risk breast cancer patients.

## 1 Introduction

BRCA mutations represent the most clinically actionable germline alterations in breast cancer. These mutations substantially increase lifetime cancer risk and influence clinical decision-making, including genetic counseling, surveillance strategies, and systemic treatment selection, such as PARP inhibitor therapy (1–5). Accordingly, contemporary clinical guidelines emphasize the importance of identifying BRCA mutation carriers among patients with suspected hereditary breast cancer. However, genetic testing remains resource-intensive and may not be universally accessible, leading to delays or underutilization even among high-risk patients. This highlights the need for practical, noninvasive approaches to prioritize patients most likely to benefit from confirmatory testing.

Breast magnetic resonance imaging (MRI) plays a central role in the evaluation and surveillance of high-risk breast cancer patients, particularly among individuals with BRCA mutations or strong familial risk (6–16). MRI provides detailed information on tumor morphology, enhancement patterns, and the surrounding breast tissue environment. Previous studies have suggested that BRCA-associated breast cancers exhibit distinctive clinicopathologic and imaging characteristics, including higher histologic grade, differences in receptor status, and specific MRI features (7–11, 17). Radiologists routinely interpret MRI findings using standardized descriptors such as those defined in the Breast Imaging Reporting and Data System (BI-RADS). These radiologist-assessed semantic features capture clinically meaningful imaging patterns that reflect tumor biology. However, radiologist interpretation may be inherently subjective and not fully capture subtle imaging patterns associated with genetic alterations.

To complement semantic MRI interpretation, radiomics has emerged as a quantitative imaging analysis approach for extracting high-dimensional features describing tumor shape, intensity distribution, and texture patterns (6, 18). Radiomics enables systematic characterization of tumor heterogeneity and has been increasingly applied in radiogenomics studies that link imaging phenotypes to genomic alterations (6, 18). Two studies have explored MRI-based radiomics to predict BRCA mutation status or hereditary breast cancer risk (19, 20). More recently, multimodal predictive models that combine ultrasound imaging-derived radiomics features with clinicopathologic variables have been proposed to improve predictive performance for identifying germline BRCA mutation (21). However, reported performance varies widely across studies, and generalizability remains a major challenge due to differences in imaging protocols, patient populations, and modeling pipelines. Importantly, most prior work has been developed and evaluated on single-institution datasets or with limited external validation, leaving uncertainty about its robustness in real-world clinical settings. Therefore, a systematic multicenter evaluation that contrasts clinicopathologic-only, radiomics-only, and multimodal predictors under both internal and cross-center external validation is needed.

In this study, we aim to evaluate the predictive value of clinicopathologic variables, radiologist-assessed MRI semantic features, and MRI-derived radiomics features for germline BRCA mutation prediction in high-risk breast cancer patients, using data from two institutions. We develop machine learning (ML) models using each modality individually and in multimodal form and evaluate their performance using both repeated internal validation and cross-center external validation. We hypothesize that integrating clinical, semantic imaging, and radiomics features would provide complementary information and improve prediction performance and generalizability across institutions.

## 2 Patients and methods

### 2.1 Patients

**Figure 1:**
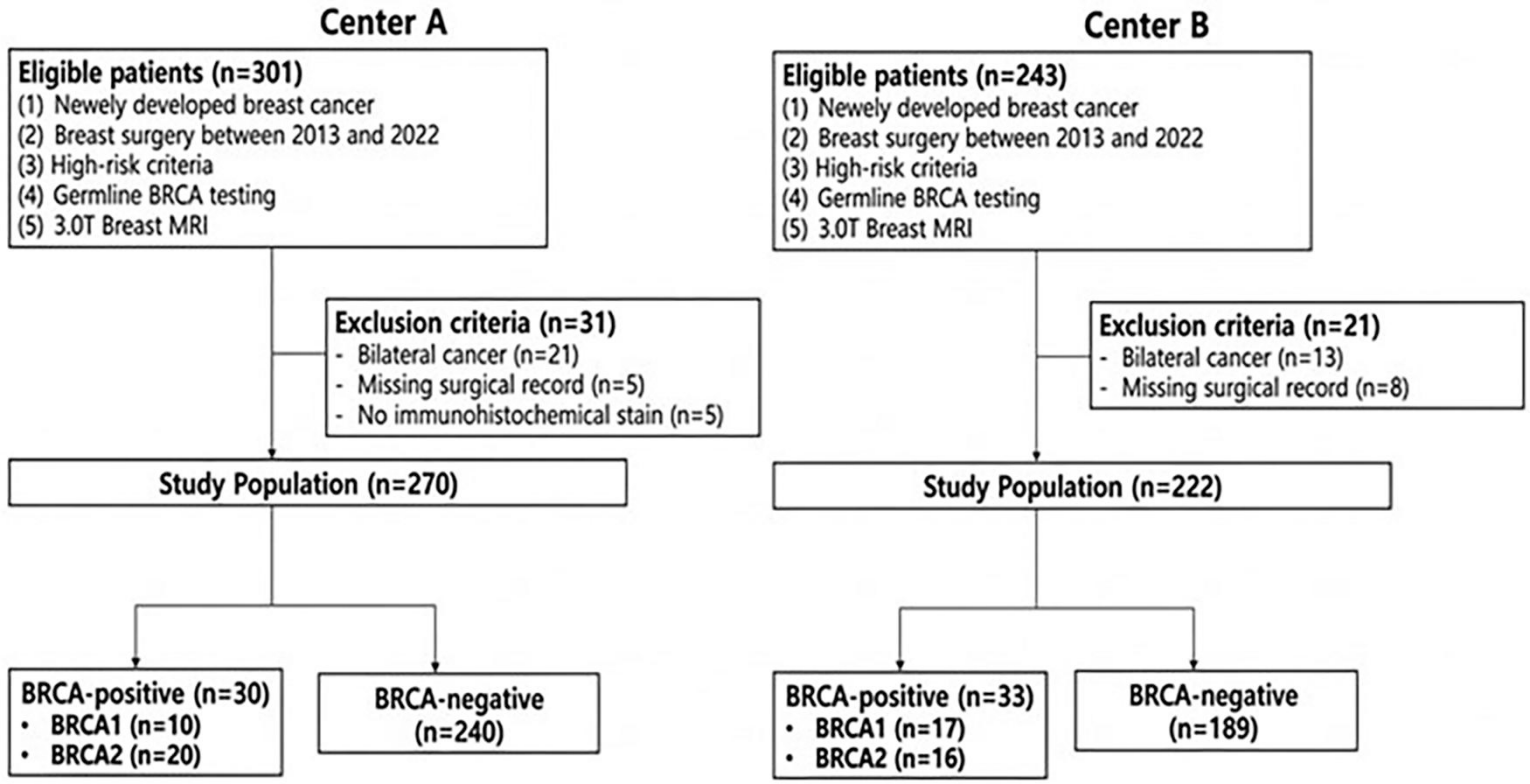
Study flowchart describing cohort construction at each center.

This retrospective multicenter study included high-risk breast cancer patients from Center A (Korea University Guro Hospital) and Center B (Korea University Anam Hospital) who underwent preoperative breast MRI and germline BRCA testing. High-risk criteria included early age at diagnosis (under 40 years), triple-negative breast cancer (TNBC), bilateral disease, and/or a family history suggestive of hereditary breast cancer. At each center, eligible patients were initially identified from two sources: (1) Patients with complete structured clinical and histopathologic variables, (2) Patients with available preoperative breast MRI suitable for radiomic feature extraction from 2-minute contrast-enhanced subtraction images (sub-T1WI), which were generated by subtracting pre-contrast series from the second post-contrast series and T2-weighted images (T2WI). Patients with bilateral breast cancer were subsequently excluded during cohort assembly because their tumors could not be analyzed with a single-tumor ROI for radiomics extraction (Figure 1).

As shown in Figure 1, the cohorts comprise centers A and B with 270 patients (BRCA+: 30; BRCA−: 240), and 222 patients (BRCA+: 33; BRCA−: 189). Institutional review board (IRB) approval was obtained, and informed consent was waived due to the retrospective study design.

### 2.2 Clinicopathologic features

Clinicopathologic variables were extracted from electronic medical records and included demographic information, histopathologic findings, and immunohistochemical markers. The clinicopathologic variables used in this study included age at diagnosis, histologic grade, lymphovascular invasion, estrogen receptor (ER) status, progesterone receptor (PR) status, Human epidermal growth factor receptor 2 (HER2) status, Ki-67 proliferation index, and molecular subtype classification (Luminal, HER2-enriched, TNBC). These variables were selected because they are routinely available in clinical practice and have been reported to be associated with hereditary breast cancer characteristics.

### 2.3 Radiologist-assessed MRI features

Radiologist-assessed MRI features were obtained from analysis of two breast radiologists (18 and 9 years of experience) in consensus, according to the 2013 BI-RADS MR lexicon (22). These semantic imaging features represent expert interpretation of tumor morphology and the surrounding breast tissue environment on sub-T1WI and T2WI. The collected radiologist-assessed MRI variables included breast fibroglandular tissue, background parenchymal enhancement, and determination of whether the lesion exhibited mass or non-mass enhancement (NME). For the masses, their shape, margin, and internal enhancement pattern were evaluated. On T2WI, intra-tumoral high signal intensity, peritumoral edema, indicated visually by higher than that of blood vessels or surrounding parenchymal tissues, was assessed. The presence of axillary lymph node enlargement was determined if meeting one or more of the following criteria: abnormal margin or shape of the lymph node, abnormal cortical thickness, or effacement of the fatty hilum (22). These features were used to represent clinically interpretable MRI characteristics evaluated by experienced breast radiologists.

### 2.4 MRI radiomics features

Two breast radiologists who were blinded to the clinicopathologic data manually drew the intratumor region-of-interest (ROI) of the tumor from the top to the bottom of the tumor for the three-dimensional segmentation of tumors with an open-source software, 3D Slicer (http://www.slicer.org) using sub-T1WI and T2WI. The ROIs manually drawn on sequential slices were rendered into a 3D volume. Radiomics features were extracted from tumor masks using PyRadiomics (version 3.0.1). To improve cross-center comparability, images were resampled to a uniform voxel spacing using interpolation, and gray-level discretization was performed using a fixed bin width. Radiomic feature definitions followed the recommendations of the Image Biomarker Standardisation Initiative (23). For the descriptive baseline tables (Tables 1, 2), one representative feature for each major family was reported to maintain readability. For the predictive machine learning analysis, all radiomics features, including shape, first-order statistics, and texture-matrix families, were used in their entirety as input to all machine learning models, without applying any dimensionality reduction or supervised feature selection.

**Table 1:** Baseline characteristics in internal and external validations.

Center A	Internal validation			External validation		
	Training (*n* = 216)	Testing (*n* = 54)	*P* value	Center A (*n* = 270)	Center B (*n* = 222)	*P* value
Clinicopathologic features						
Age at diagnosis	45.00 [39.00, 53.00]	48.00 [37.25, 56.00]	0.438	45.50 [39.00, 53.00]	44.50 [37.00, 54.75]	0.452
Age at diagnosis ≤40 years	78 (36.11%)	19 (35.19%)	1.000	97 (35.93%)	91 (40.99%)	0.264
Histologic grade 3	100 (46.30%)	24 (44.44%)	0.879	124 (45.93%)	92 (41.44%)	0.361
Ki-67 index ≥14%	30 (13.89%)	7 (12.96%)	1.000	37 (13.70%)	131 (59.01%)	<0.001
Lymphovascular invasion	45 (20.83%)	5 (9.26%)	0.052	50 (18.52%)	46 (20.72%)	0.569
ER positivity	151 (69.91%)	43 (79.63%)	0.178	194 (71.85%)	153 (68.92%)	0.488
PR positivity	153 (70.83%)	42 (77.78%)	0.396	195 (72.22%)	153 (68.92%)	0.428
HER2 positivity	31 (14.35%)	12 (22.22%)	0.210	43 (15.93%)	51 (22.97%)	0.051
Molecular subtypes						
Luminal	162 (75.00%)	46 (85.19%)	0.147	208 (77.04%)	163 (73.42%)	0.400
HER2-enriched	7 (3.24%)	2 (3.70%)	1.000	9 (3.33%)	14 (6.31%)	0.136
TNBC	47 (21.76%)	6 (11.11%)	0.087	53 (19.63%)	45 (20.27%)	0.910
Radiologist-assessed MRI semantic features						
Non-mass enhancement	8 (3.70%)	3 (5.56%)	0.464	11 (4.07%)	13 (5.86%)	0.404
Dense fibroglandular tissue	179 (82.87%)	44 (81.48%)	0.841	223 (82.59%)	182 (81.98%)	0.906
Minimal or mild background parenchymal enhancement	93 (43.06%)	29 (53.70%)	0.172	122 (45.19%)	119 (53.60%)	0.070
Intratumoral high signal intensity on T2-weighted images	58 (26.85%)	15 (27.78%)	0.866	73 (27.04%)	115 (51.80%)	<0.001
Peritumoral edema on T2-weighted images	166 (76.85%)	41 (75.93%)	0.859	207 (76.67%)	146 (65.77%)	0.009
Axillary lymph node enlargement	75 (34.72%)	23 (42.59%)	0.342	98 (36.30%)	117 (52.70%)	<0.001
Radiomics features (sub-T1WI)						
Shape sphericity	0.68 [0.60, 0.74]	0.71 [0.64, 0.74]	0.342	0.68 [0.60, 0.74]	0.67 [0.58, 0.74]	0.244
Shape voxel volume	937.00 [389.41, 2,150.87]	891.25 [376.61, 1,862.98]	0.882	925.22 [385.39, 2,093.82]	2,737.59 [1,177.02, 7,220.12]	<0.001
First-order entropy	0.12 [0.06, 0.27]	0.10 [0.06, 0.29]	0.802	0.11 [0.06, 0.27]	0.11 [0.05, 0.18]	0.073
GLCM contrast	0.02 [0.01, 0.05]	0.01 [0.01, 0.06]	0.795	0.02 [0.01, 0.05]	0.01 [0.01, 0.03]	0.042
GLRLM run entropy	3.50 [2.99, 3.79]	3.39 [3.03, 3.73]	0.772	3.48 [3.01, 3.78]	3.73 [3.35, 4.07]	<0.001
Radiomics features (T2WI)						
Shape sphericity	0.53 [0.47, 0.57]	0.54 [0.49, 0.58]	0.171	0.53 [0.47, 0.57]	0.57 [0.52, 0.63]	<0.001
Shape voxel volume	4,411.46 [1,837.90, 10,039.85]	4,045.94 [1,912.44, 8,293.76]	0.618	4,337.64 [1,862.56, 9,696.85]	6,951.98 [2,745.62, 20,566.83]	<0.001
First-order entropy	0.03 [−0.00, 0.11]	0.04 [−0.00, 0.11]	0.675	0.03 [−0.00, 0.11]	0.02 [−0.00, 0.06]	0.003
GLCM contrast	0.00 [0.00, 0.02]	0.01 [0.00, 0.02]	0.676	0.00 [0.00, 0.02]	0.00 [0.00, 0.01]	<0.001
GLRLM run entropy	3.33 [2.90, 3.65]	3.22 [2.95, 3.67]	0.505	3.30 [2.91, 3.66]	3.50 [3.09, 3.88]	<0.001

**Table 2:** Univariable and multivariable analysis of germline BRCA mutation in relation to clinicopathologic, radiologist-assessed MRI semantic, and radiomic features in the training set of Center A.

Center A	Univariable OR	*P* value	Multivariable OR	*P* value
Clinicopathologic features				
Histologic grade 3	3.19 (1.26, 8.05)	0.014	2.49 (0.86, 7.21)	0.093
Ki-67 index ≥14%	0.87 (0.24, 3.13)	0.835		
Age at diagnosis	1.00 (0.96, 1.04)	0.969		
Lymphovascular invasion	1.00 (0.35, 2.84)	1.000		
ER positivity	0.38 (0.16, 0.90)	0.028	0.61 (0.23, 1.65)	0.332
PR positivity	0.44 (0.19, 1.04)	0.062		
HER2 positivity	1.68 (0.58, 4.89)	0.341		
Molecular subtypes				
Luminal	0.51 (0.21, 1.24)	0.139		
HER2-enriched	3.40 (0.62, 18.58)	0.158		
TNBC	1.56 (0.61, 4.03)	0.354		
Radiologist-assessed MRI semantic features				
Non-mass enhancement	N/A	1.000		
Dense fibroglandular tissue	1.04 (0.33, 3.24)	0.949		
Minimal or mild background parenchymal enhancement	0.94 (0.40, 2.22)	0.884		
Intratumoral high signal intensity on T2-weighted images	0.90 (0.34, 2.38)	0.828		
Peritumoral edema on T2-weighted images	3.67 (0.83, 16.17)	0.086		
Axillary lymph node enlargement	0.75 (0.30, 1.90)	0.545		
Radiomics features (sub-T1WI)				
Shape sphericity	0.51 (0.01, 26.40)	0.738		
Shape voxel volume	1.00 (1.00, 1.00)	0.757		
First-order entropy	1.72 (0.22, 13.62)	0.606		
GLCM contrast	2.41 (0.00, 10,492.79)	0.837		
GLRLM run entropy	1.88 (0.90, 3.95)	0.093		
Radiomics features (T2WI)				
Shape sphericity	7.54 (0.03, 2,069.95)	0.481		
Shape voxel volume	1.00 (1.00, 1.00)	0.831		
First-order entropy	0.03 (0.00, 4.72)	0.174		
GLCM contrast	0.00 (0.00, 3,424.50)	0.222		
GLRLM run entropy	1.32 (0.67, 2.58)	0.418		

### 2.5 Statistical analysis

Statistical analyses were performed to summarize patient characteristics and assess associations between germline BRCA mutation status and clinicopathologic variables, radiologist-assessed semantic features, and MRI-derived radiomics features. Continuous variables are presented as medians with interquartile ranges and were compared using the Wilcoxon rank-sum test, while categorical variables are presented as counts (percentages) and were compared using the chi-square test or Fisher's exact test, as appropriate. To identify features associated with germline BRCA mutation, univariate logistic regression was first performed, and variables with *P* < 0.05 were entered into multivariate analysis. Statistical analyses were conducted using SPSS version 20.0, R version 3.5.1, and Python version 3.7.4.

### 2.6 Predictive machine learning analysis

Predictive modeling was performed using six machine learning algorithms to evaluate the predictive performance of clinicopathologic variables, radiologist-assessed MRI features, and MRI-derived radiomics features. We applied logistic regression (LR), random forests (RF), support vector machines (SVMs) with radial basis function kernels, gradient-boosted models (LightGBM and XGBoost), and a multilayer perceptron (MLP). The LR model was used as a baseline linear model because of its interpretability and clinical relevance. Tree-based ensemble methods, boosting models, and the MLP were expected to capture nonlinear relationships and feature interactions. All models were implemented using standardized libraries and consistent preprocessing pipelines across feature sets to ensure a fair comparison. Because the dataset was imbalanced between BRCA mutation-positive and mutation-negative cases, class weights were applied during model training to mitigate bias toward the majority class.

Internal validation was performed to assess model stability and the average predictive performance in Center A. For each experiment, the dataset was randomly split into training (80%) and testing (20%) subsets. This process was repeated 10 times with different random seeds, generating 10 independent train-test splits. For each split, models were trained on the training set and evaluated on the corresponding test set. Performance metrics were averaged across repetitions, and standard deviations were reported to reflect variability due to data partitioning. This repeated random-split strategy was chosen to reduce dependence on a single split and provide a more robust estimate of model performance.

External validation was then performed to assess cross-institutional generalizability. Models were trained exclusively on data from Center A and evaluated on data from Center B. Model fitting was repeated with different random seeds to quantify optimization variability, such as weight initialization and stochastic training, rather than to create independent test samples.

Model performance was primarily evaluated using the area under the receiver operating characteristic curve (AUC). For all experiments, results are reported as mean AUC with corresponding standard deviation. In addition to AUROC, we computed the area under the precision–recall curve (AUPRC), which is more informative than AUROC under marked class imbalance, as well as sensitivity, specificity, and *F*1-score at the optimal classification cutoff determined by Youden's *J* statistic on the training set. AUROC was used as the primary metric for model comparison, with the remaining metrics provided as complementary indicators of calibration and operating-point behavior.

### 2.7 Shapley additive explanations (SHAP) analysis

To improve the interpretability of the machine learning models, SHAP was applied to quantify each feature's contribution to model predictions (24, 32). SHAP values are derived from cooperative game theory and represent the marginal contribution of each feature by considering all possible feature combinations. SHAP summary plots were generated using the test dataset to visualize the distribution of feature contributions across samples. Each point represents an individual patient, with the horizontal axis indicating the magnitude and direction of the contribution and the color representing the feature value. Positive SHAP values indicate increased probability of germline BRCA mutation, whereas negative values indicate decreased probability. To enable direct comparison, identical feature sets and preprocessing pipelines were used across models. SHAP analysis was performed on the best-balanced LR models in each validation setting, selected based on combined consideration of AUROC, AUPRC, sensitivity, and *F*1-score.

## 3 Results

### 3.1 Patient characteristics

Table 1 summarizes the baseline characteristics of patients in both the internal validation scenario (Center A training vs. testing split) and the external validation scenario (Center A vs. Center B). Overall, most clinicopathologic and MRI-derived variables were well balanced between the internal training and testing subsets, indicating that the random split introduced no major baseline differences.

When comparing two centers, the Ki-67 index ≥14%, intra-tumoral high signal intensity and peritumoral edema on T2WI, and axillary lymph node enlargement differed between the two centers (*P* < 0.05). In addition, several radiomics features showed significant differences across centers (*P* < 0.05). These findings suggest potential variations in patient populations and imaging characteristics across institutions, highlighting the importance of external validation to assess model generalizability.

### 3.2 Associations between clinicopathologic, radiologist-assessed MRI semantic features, MRI radiomics features, and BRCA mutation

Table 2 presents the results of the univariable and multivariable logistic regression analyses evaluating the associations between clinicopathologic variables, radiologist-assessed MRI semantic features, MRI radiomics features, and germline BRCA mutation status in the training cohort of Center A.

In the univariable analysis, histologic grade 3 [odds ratio (OR) 3.189, *P* = 0.014] and lower estrogen receptor positivity (OR 0.381, *P* = 0.028) were significantly associated with BRCA mutation status. Most radiologist-assessed MRI features and radiomics features were not significantly associated with BRCA mutation status in the univariable analysis.

In the multivariable analysis, none of the variables remained statistically significant. However, histologic grade 3 showed a borderline association with BRCA mutation status (OR 2.49, *P* = 0.093), suggesting a potential trend toward higher BRCA mutation risk in high-grade tumors.

### 3.3 Internal validations

Table 3 summarizes the internal validation results for BRCA mutation prediction using unimodal models and their multimodal combinations. Among the unimodal models, the clinicopathologic-based LR model showed the highest AUROC of 0.73, and the radiologist-assessed semantic feature-based models showed the lowest AUROC scores, ranging from 0.40 to 0.54. In addition, radiomics derived from sub-T1WI-based gradient-boosting models, XGBoost and LightGBM, also achieved comparable performance, with AUROCs of 0.72 and 0.71, respectively. Beyond AUROC, AUPRC values were generally low across all models (≤0.40), and cutoff-based metrics (sensitivity, specificity, *F*1-score) reflected the strong class imbalance, with several radiomics-only and multimodal combinations collapsing to a degenerate operating point (sensitivity or specificity near 0).

**Table 3:** Diagnostic performance of 6 machine learning models in internal validations of Center A.

Metric	Model	CL	RA	CL + RA	RD(T2WI)	RD (sub-T1WI)	CL + RA + RD (T2WI)	CL + RA + RD (sub-T1WI)
AUROC	LightGBM	0.64 (0.08)	0.40 (0.06)	0.63 (0.07)	0.64 (0.08)	0.71 (0.09)	0.62 (0.10)	0.63 (0.07)
	LR	0.73 (0.08)	0.54 (0.08)	0.64 (0.08)	0.59 (0.09)	0.65 (0.09)	0.62 (0.10)	0.60 (0.15)
	MLP	0.58 (0.16)	0.49 (0.10)	0.53 (0.13)	0.64 (0.12)	0.69 (0.08)	0.63 (0.10)	0.66 (0.10)
	RF	0.67 (0.07)	0.41 (0.08)	0.63 (0.07)	0.61 (0.09)	0.68 (0.08)	0.61 (0.09)	0.67 (0.10)
	SVM	0.63 (0.17)	0.51 (0.11)	0.51 (0.17)	0.47 (0.20)	0.67 (0.10)	0.48 (0.17)	0.57 (0.07)
	XGBoost	0.66 (0.11)	0.42 (0.07)	0.63 (0.07)	0.62 (0.11)	0.72 (0.12)	0.62 (0.11)	0.63 (0.09)
AUPRC	LightGBM	0.21 (0.12)	0.09 (0.01)	0.21 (0.12)	0.16 (0.05)	0.26 (0.15)	0.15 (0.03)	0.17 (0.05)
	LR	0.40 (0.19)	0.24 (0.16)	0.32 (0.18)	0.13 (0.03)	0.18 (0.07)	0.15 (0.04)	0.14 (0.05)
	MLP	0.15 (0.05)	0.12 (0.06)	0.17 (0.09)	0.19 (0.07)	0.28 (0.11)	0.17 (0.05)	0.17 (0.07)
	RF	0.16 (0.03)	0.10 (0.03)	0.17 (0.10)	0.16 (0.07)	0.21 (0.07)	0.18 (0.08)	0.22 (0.11)
	SVM	0.18 (0.10)	0.16 (0.11)	0.11 (0.03)	0.15 (0.10)	0.25 (0.06)	0.16 (0.10)	0.17 (0.10)
	XGBoost	0.19 (0.10)	0.10 (0.04)	0.20 (0.13)	0.15 (0.05)	0.24 (0.12)	0.17 (0.07)	0.18 (0.06)
Sensitivity	LightGBM	0.22 (0.18)	0.37 (0.20)	0.18 (0.17)	0.00 (0.00)	0.00 (0.00)	0.00 (0.00)	0.00 (0.00)
	LR	0.88 (0.16)	0.50 (0.28)	0.63 (0.30)	0.18 (0.28)	0.12 (0.18)	0.22 (0.29)	0.13 (0.29)
	MLP	0.42 (0.31)	0.47 (0.20)	0.20 (0.17)	0.00 (0.00)	0.13 (0.17)	0.00 (0.00)	0.03 (0.07)
	RF	0.35 (0.25)	0.37 (0.13)	0.10 (0.18)	0.00 (0.00)	0.00 (0.00)	0.00 (0.00)	0.00 (0.00)
	SVM	0.68 (0.30)	0.33 (0.27)	0.35 (0.31)	0.05 (0.11)	0.05 (0.11)	0.02 (0.05)	0.03 (0.07)
	XGBoost	0.32 (0.21)	0.33 (0.16)	0.23 (0.25)	0.00 (0.00)	0.00 (0.00)	0.00 (0.00)	0.00 (0.00)
Specificity	LightGBM	0.79 (0.07)	0.54 (0.10)	0.89 (0.07)	1.00 (0.00)	1.00 (0.00)	1.00 (0.00)	1.00 (0.00)
	LR	0.57 (0.07)	0.56 (0.21)	0.60 (0.11)	0.87 (0.18)	0.90 (0.09)	0.86 (0.18)	0.91 (0.11)
	MLP	0.73 (0.06)	0.58 (0.11)	0.83 (0.06)	0.98 (0.03)	0.95 (0.06)	0.99 (0.01)	0.95 (0.04)
	RF	0.81 (0.06)	0.54 (0.09)	0.91 (0.09)	1.00 (0.00)	1.00 (0.00)	1.00 (0.00)	1.00 (0.00)
	SVM	0.59 (0.24)	0.65 (0.22)	0.65 (0.26)	0.98 (0.05)	0.98 (0.03)	0.99 (0.04)	0.96 (0.05)
	XGBoost	0.81 (0.08)	0.57 (0.07)	0.86 (0.09)	1.00 (0.00)	1.00 (0.00)	1.00 (0.00)	1.00 (0.00)
*F*1 score	LightGBM	0.15 (0.13)	0.14 (0.07)	0.17 (0.15)	0.00 (0.00)	0.00 (0.00)	0.00 (0.00)	0.00 (0.00)
	LR	0.33 (0.06)	0.18 (0.10)	0.25 (0.09)	0.11 (0.12)	0.10 (0.14)	0.12 (0.14)	0.07 (0.16)
	MLP	0.22 (0.15)	0.19 (0.09)	0.15 (0.14)	0.00 (0.00)	0.15 (0.19)	0.00 (0.00)	0.03 (0.07)
	RF	0.22 (0.13)	0.15 (0.05)	0.07 (0.12)	0.00 (0.00)	0.00 (0.00)	0.00 (0.00)	0.00 (0.00)
	SVM	0.27 (0.08)	0.13 (0.10)	0.13 (0.11)	0.05 (0.13)	0.06 (0.13)	0.02 (0.05)	0.04 (0.09)
	XGBoost	0.22 (0.15)	0.14 (0.06)	0.16 (0.15)	0.00 (0.00)	0.00 (0.00)	0.00 (0.00)	0.00 (0.00)

Values are presented as mean (standard deviation) across 10 repeated random train–test splits in Center A. To keep the table font size consistent given the number of reported metrics, all values are rounded to two decimal places. AUROC, area under the receiver operating characteristic curve; AUPRC, area under the precision–recall curve. Sensitivity, specificity, and *F*1-score were computed at the optimal cutoff determined by Youden's *J* statistic on the training set. CL, clinicopathologic features; RA, radiologist-assessed MRI semantic features; RD (T2WI), radiomics from T2-weighted images; RD (sub-T1WI), radiomics from 2-minute contrast-enhanced subtraction images.

### 3.4 External validation

Table 4 presents the external validation results obtained by training models on Center A and testing them on Center B. Among the unimodal models, the clinicopathologic RF model demonstrated the highest AUROC of 0.73, whereas radiologist-assessed semantic models showed lower AUROCs, ranging from 0.54 to 0.58. Radiomics-only models also demonstrated lower external performance, ranging from 0.49 to 0.62. The combined clinicopathologic and radiologist-assessed RF model achieved the highest overall external performance with an AUROC of 0.77, and the multimodal LR model integrating sub-T1WI radiomics features achieved a comparable AUROC of 0.72.

**Table 4:** Diagnostic performance of 6 machine learning models in external validations of Center B.

Metric	Model	CL	RA	CL + RA	RD (T2WI)	RD (sub-T1WI)	CL + RA + RD (T2WI)	CL + RA + RD (sub-T1WI)
AUROC	LightGBM	0.66 (0.01)	0.58 (0.01)	0.69 (0.01)	0.51 (0.02)	0.61 (0.02)	0.51 (0.02)	0.62 (0.01)
	LR	0.68 (0.00)	0.57 (0.01)	0.69 (0.01)	0.49 (0.01)	0.59 (0.04)	0.55 (0.07)	0.72 (0.00)
	MLP	0.51 (0.04)	0.56 (0.02)	0.64 (0.02)	0.55 (0.03)	0.57 (0.02)	0.56 (0.04)	0.60 (0.03)
	RF	0.73 (0.01)	0.55 (0.02)	0.77 (0.01)	0.49 (0.02)	0.59 (0.02)	0.49 (0.01)	0.59 (0.01)
	SVM	0.71 (0.01)	0.54 (0.09)	0.74 (0.03)	0.53 (0.04)	0.55 (0.02)	0.53 (0.04)	0.54 (0.03)
	XGBoost	0.71 (0.02)	0.56 (0.01)	0.72 (0.01)	0.52 (0.03)	0.62 (0.01)	0.55 (0.02)	0.62 (0.01)
AUPRC	LightGBM	0.28 (0.01)	0.20 (0.00)	0.28 (0.03)	0.15 (0.01)	0.20 (0.01)	0.15 (0.01)	0.20 (0.00)
	LR	0.51 (0.00)	0.25 (0.01)	0.50 (0.05)	0.14 (0.01)	0.19 (0.01)	0.17 (0.03)	0.28 (0.00)
	MLP	0.17 (0.02)	0.20 (0.04)	0.28 (0.01)	0.20 (0.03)	0.21 (0.02)	0.20 (0.02)	0.23 (0.02)
	RF	0.27 (0.01)	0.17 (0.02)	0.31 (0.01)	0.16 (0.01)	0.22 (0.02)	0.16 (0.01)	0.21 (0.01)
	SVM	0.32 (0.03)	0.22 (0.08)	0.36 (0.02)	0.17 (0.02)	0.19 (0.02)	0.17 (0.01)	0.20 (0.02)
	XGBoost	0.28 (0.03)	0.20 (0.01)	0.33 (0.02)	0.16 (0.01)	0.21 (0.01)	0.18 (0.01)	0.22 (0.01)
Sensitivity	LightGBM	0.19 (0.03)	0.52 (0.00)	0.14 (0.03)	0.00 (0.00)	0.00 (0.00)	0.00 (0.00)	0.00 (0.00)
	LR	0.73 (0.00)	0.48 (0.00)	0.72 (0.02)	0.05 (0.07)	0.28 (0.19)	0.15 (0.19)	0.39 (0.00)
	MLP	0.20 (0.06)	0.49 (0.01)	0.26 (0.03)	0.02 (0.02)	0.05 (0.05)	0.01 (0.02)	0.04 (0.03)
	RF	0.51 (0.08)	0.48 (0.01)	0.03 (0.08)	0.00 (0.00)	0.00 (0.00)	0.00 (0.00)	0.00 (0.00)
	SVM	0.43 (0.12)	0.48 (0.30)	0.46 (0.23)	0.00 (0.00)	0.00 (0.00)	0.00 (0.00)	0.00 (0.00)
	XGBoost	0.25 (0.08)	0.48 (0.00)	0.16 (0.05)	0.00 (0.00)	0.00 (0.00)	0.00 (0.00)	0.00 (0.00)
Specificity	LightGBM	0.90 (0.02)	0.51 (0.00)	0.96 (0.02)	1.00 (0.00)	1.00 (0.00)	1.00 (0.00)	1.00 (0.00)
	LR	0.64 (0.00)	0.57 (0.00)	0.65 (0.02)	0.93 (0.05)	0.86 (0.11)	0.90 (0.07)	0.87 (0.00)
	MLP	0.88 (0.02)	0.53 (0.00)	0.94 (0.01)	0.99 (0.01)	0.98 (0.02)	1.00 (0.01)	0.99 (0.01)
	RF	0.80 (0.03)	0.54 (0.02)	0.98 (0.02)	1.00 (0.00)	1.00 (0.00)	1.00 (0.00)	1.00 (0.00)
	SVM	0.83 (0.06)	0.57 (0.27)	0.85 (0.11)	1.00 (0.00)	1.00 (0.00)	1.00 (0.00)	1.00 (0.00)
	XGBoost	0.90 (0.02)	0.54 (0.02)	0.95 (0.02)	1.00 (0.00)	1.00 (0.00)	1.00 (0.00)	1.00 (0.00)
*F*1 score	LightGBM	0.22 (0.02)	0.24 (0.00)	0.20 (0.03)	0.00 (0.00)	0.00 (0.00)	0.00 (0.00)	0.00 (0.00)
	LR	0.38 (0.00)	0.24 (0.00)	0.39 (0.00)	0.05 (0.06)	0.23 (0.12)	0.13 (0.13)	0.37 (0.00)
	MLP	0.21 (0.05)	0.23 (0.00)	0.32 (0.02)	0.03 (0.04)	0.08 (0.08)	0.02 (0.04)	0.08 (0.06)
	RF	0.38 (0.03)	0.23 (0.00)	0.03 (0.09)	0.00 (0.00)	0.00 (0.00)	0.00 (0.00)	0.00 (0.00)
	SVM	0.36 (0.03)	0.21 (0.11)	0.37 (0.07)	0.00 (0.00)	0.00 (0.00)	0.00 (0.00)	0.00 (0.00)
	XGBoost	0.26 (0.06)	0.24 (0.01)	0.22 (0.05)	0.00 (0.00)	0.00 (0.00)	0.00 (0.00)	0.00 (0.00)

Values are presented as mean (standard deviation) across repeated model fits trained on Center A and evaluated on Center B. To keep the table font size consistent given the number of reported metrics, all values are rounded to two decimal places. A degenerate operating point (sensitivity ≈ 0, specificity ≈ 1, *F*1 ≈ 0) reflects models that classified nearly all external cases as mutation-negative under cross-center distribution shift. AUROC, area under the receiver operating characteristic curve; AUPRC, area under the precision–recall curve. CL, clinicopathologic features; RA, radiologist-assessed MRI semantic features; RD (T2WI), radiomics from T2-weighted images; RD (sub-T1WI), radiomics from 2-minute contrast-enhanced subtraction images.

Consistent with the AUROC results, cutoff-based metrics confirmed the limited transferability of radiomics features: radiomics-only and several multimodal combinations frequently collapsed to a degenerate operating point in external validation, whereas the clinicopathologic and combined clinicopathologic and radiologist-assessed models retained more balanced sensitivity and specificity.

These results indicate that clinicopathologic and radiologist-assessed MRI features exhibit greater cross-institutional robustness than radiomics features, although sub-T1WI-based radiomics features may provide complementary information in specific settings.

### 3.5 SHAP analysis

**Figure 2:**
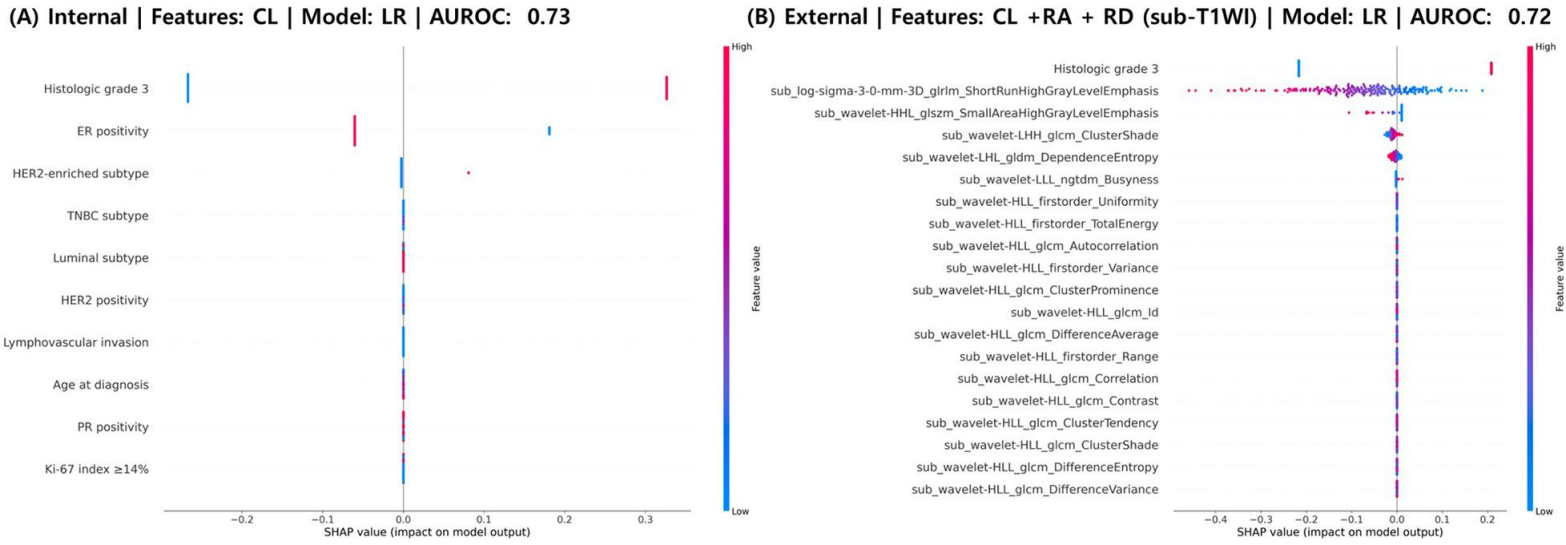
SHAP summary plots for the best-balanced LR models in internal and external validation. **(A)** Internal validation: SHAP summary plot for the clinicopathologic LR model (AUROC = 0.73, sensitivity = 0.88, *F*1 = 0.33). Histologic grade 3 and ER positivity showed the broadest and most consistent SHAP value distributions across samples. **(B)** External validation: SHAP summary plot for the multimodal LR model integrating clinicopathologic, radiologist-assessed MRI, and sub-T1WI radiomics features (AUROC = 0.72, sensitivity = 0.39, *F*1 = 0.37). Histologic grade 3 and one sub-T1WI radiomics feature (sub_log-sigma-3-0-mm-3D_glrlm_ShortRunHighGrayLevelEmphasis) showed the broadest and most consistent contributions. In both panels, each point represents an individual patient, with the horizontal axis indicating the magnitude and direction of the SHAP contribution and the color indicating the feature value (red, high; blue, low). Positive SHAP values indicate increased predicted probability of germline BRCA mutation.

As shown in Figure 2, SHAP analysis was performed on the best-balanced LR models in each validation setting. The LR models were selected because they achieved the highest combined performance across AUROC, AUPRC, sensitivity, and *F*1-score.

For internal validation (Figure 2A), the clinicopathologic LR model (AUROC = 0.73, sensitivity = 0.88, *F*1 = 0.33) was analyzed. The SHAP summary plot showed that histologic grade 3 was the most influential feature, with high grade associated with an increased predicted probability of BRCA mutation. ER positivity was the second most influential feature, with low ER expression contributing to a higher predicted probability. Remaining variables, including HER2-enriched subtype, TNBC subtype, Luminal subtype, HER2 positivity, lymphovascular invasion, age at diagnosis, PR positivity, and Ki-67 index, showed minimal SHAP contributions. This indicates that internal predictions were primarily driven by histologic grade and ER status.

For external validation (Figure 2B), the multimodal LR model integrating clinicopathologic, radiologist-assessed MRI, and sub-T1WI radiomics features (AUROC = 0.72, sensitivity = 0.39, *F*1 = 0.37) was analyzed. Histologic grade 3 remained a dominant feature with a clear directional effect, while one sub-T1WI radiomics feature, a GLRLM short-run high gray-level emphasis derived from a Laplacian-of-Gaussian filtered image (*σ* = 3.0 mm; original PyRadiomics name: sub_log-sigma-3-0-mm-3D_glrlm_ShortRunHighGrayLevelEmphasis), showed a broad and consistent SHAP distribution across samples. Additional sub-T1WI radiomics features derived from wavelet decompositions (a GLSZM small-area high gray-level emphasis, a GLCM cluster shade, and a GLDM dependence entropy) contributed more modestly. The full PyRadiomics feature names are shown in Figure 2B. The stable directional effects observed across samples reflect the additive nature of the LR model and support its robustness and interpretability for cross-center deployment.

## 4 Discussion and conclusion

In this multicenter study, we evaluated whether clinicopathologic variables, radiologist-assessed MRI semantic features, and MRI-derived radiomic features could predict germline BRCA mutation status in high-risk breast cancer patients. Among the unimodal models, the clinicopathologic model showed the most consistent performance, with AUCs in the 0.6–0.7 range in both internal and external validation, whereas the radiologist-assessed semantic model showed the lowest performance, with AUCs in the 0.4–0.5 range. Among the multimodal combinations, the LR model integrating clinicopathologic, radiologist-assessed MRI, and sub-T1WI radiomics features achieved an external AUROC of 0.72, comparable to the highest unimodal (clinicopathologic RF, 0.73) and bimodal (clinicopathologic + radiologist-assessed RF, 0.77) models, highlighting the value of combining complementary clinical and imaging features for cross-center deployment.

These findings suggest that baseline clinicopathologic information provides a relatively stable predictive framework, whereas qualitative MRI features alone may be insufficient to capture the imaging phenotype of BRCA-mutated tumors across institutions.

Regarding MRI semantic features, several studies have reported that BRCA mutation carriers tend to show distinctive MRI findings, including morphologic features such as round shape, circumscribed margins, rim enhancement, posterior location, and high T2 signal intensity (7–11, 17). However, in our cohort, no MRI semantic feature showed a clear association with BRCA-positive status, indicating that qualitative radiologist-assessed descriptors alone may not reliably distinguish BRCA-mutated tumors.

In contrast, radiomics-based models showed stronger performance in internal validation, particularly when derived from sub-T1WI rather than T2WI, suggesting that subtraction images may capture more informative quantitative features related to BRCA mutation status. This is consistent with previous radiomics studies showing that subtraction-based MRI features can outperform features extracted from the original image series or from T2WI, likely because subtraction images better emphasize enhancement-related tumor heterogeneity (25). Previous work using quantitative MRI for BRCA prediction has mainly focused on manually extracted texture features or CAD-based kinetic descriptors, and only a limited number of studies are available (19, 20). In our study, the use of radiomic features extends this literature by leveraging higher-dimensional imaging information that may not be fully represented by conventional texture analysis alone. Although radiomics-only models performed less consistently in external validation, their internal performance indicates that quantitative imaging features can contribute meaningful information, while also underscoring their sensitivity to inter-institutional variability in acquisition, reconstruction, and segmentation.

Complex non-linear models exhibited limited cross-center generalization. For example, sub-T1WI radiomics-based MLP, XGBoost, and LightGBM achieved internal AUROCs of 0.69, 0.72, and 0.71, but dropped to 0.57, 0.62, and 0.61 in external validation (Tables 3, 4). However, the simpler linear LR model showed the most stable cross-center performance (external multimodal AUROC = 0.72). In small and imbalanced datasets like ours, simpler models are often more reliable when applied to a new institution. For this reason, model selection should be guided by external validation rather than by internal performance alone.

From a practical standpoint, our findings also place the performance of the mpMRI-based models in context with established tools such as BRCAPRO and BOADICEA, which have reported AUCs in the range of approximately 0.72–0.80 (26–28). In that regard, the multimodal MRI-based approach showed comparable discriminative ability while offering a noninvasive, imaging-based alternative that may help prioritize patients for confirmatory BRCA testing and genetic counseling in the preoperative setting. From a clinical perspective, these findings support the potential role of noninvasive machine learning-based risk stratification for prioritizing patients who may benefit most from confirmatory BRCA testing and genetic counseling. This may be particularly useful in settings where genetic testing is costly, unavailable, or delayed. A model that combines readily available clinical information with interpretable MRI features could serve as a practical decision-support tool in the preoperative setting.

Our findings align with recent work on machine learning and explainable AI in breast imaging. Yuan et al. applied SHAP-based interpretation to predict neoadjuvant chemotherapy response and long-term outcomes in breast cancer (29). Sha et al. integrated deep learning features from mammography with SHAP values to predict DCIS recurrence after lumpectomy (30). Yuan et al. further combined deep feature extraction with MRI radiomics for survival prediction following neoadjuvant chemotherapy (31). In contrast, our study targets germline BRCA mutation prediction and provides multicenter external validation with a head-to-head comparison of unimodal, bimodal, and trimodal feature combinations under a unified interpretable framework.

This study has several limitations. First, although external validation was performed, the number of participating institutions was limited, and broader multicenter validation is needed to further establish generalizability. Second, the sample size, especially the BRCA-positive subgroup, was relatively modest, which may limit model stability and the precision of performance estimates. Third, the radiomics-only models showed limited generalizability across centers, as reflected by the significant cross-center differences in radiomics features (Table 1) and the substantial drop in external validation performance. This problem can be attributed to a domain shift due to differences in scanner protocols between the two institutions. Future studies should incorporate image harmonization techniques such as ComBat, together with prospective acquisition standardization, to further improve cross-center transferability. More broadly, complementary strategies, including image acquisition standardization, intensity normalization, and radiomic feature harmonization, can collectively address domain shift and facilitate real-world clinical translation. Supervised feature selection methods, such as LASSO, mRMR, or stability selection, within nested cross-validation could also be used to improve the parsimony and cross-center robustness of radiomics-based models.

In conclusion, this multicenter study suggests that clinicopathologic variables and radiologist-assessed MRI semantic features provide robust and transferable information for predicting germline BRCA mutation status in high-risk breast cancer patients. MRI-derived radiomic features may offer additional complementary value, but their performance is less consistent across institutions. These findings support multimodal, noninvasive risk stratification as a promising approach to identify patients who may benefit from confirmatory genetic testing and counseling.

## Data Availability

The raw data supporting the conclusions of this article will be made available by the authors, without undue reservation.
